# An *Acinetobacter baumannii* acyltransferase represses pilin production to maintain energy homeostasis and overcome nutritional immunity

**DOI:** 10.21203/rs.3.rs-10119738/v1

**Published:** 2026-06-30

**Authors:** Dillon E. Kunkle, Owen S. Burroughs, Christopher J. Corcoran, Eric P. Skaar

**Affiliations:** 1 Department of Pathology, Microbiology, and Immunology, Vanderbilt University Medical Center, Nashville, Tennessee, USA; 2 Vanderbilt Institute for Infection, Immunology, and Inflammation, Vanderbilt University Medical Center, Nashville, Tennessee, USA

## Abstract

Respiratory infections are the fourth leading cause of death globally. *Acinetobacter baumannii* is a significant cause of ventilator-associated pneumonia and antibiotic-resistant attributable deaths worldwide. Iron is an essential nutrient; vertebrates exploit this by sequestering iron from invading pathogens in a process termed nutritional immunity. The competition for iron is a crucial determinant of *A. baumannii* infection severity and outcome. Here, we have discovered an *A. baumannii* acyltransferase that we have named AimS. AimS promotes fitness in low iron environments and contributes to *A. baumannii* surviving nutritional immunity encountered during pneumonia. Mechanistic studies revealed that AimS impacts fitness through maintenance of cellular ATP via repression of pili production and biofilm formation, key virulence phenotypes, through a transcriptional regulator we have named CsuR. These findings reveal an energy sparing mechanism that *A. baumannii* employs to regulate pathogenesis and ensure energy availability, enabling the pathogen to overcome nutritional immunity at the host-pathogen interface.

## INTRODUCTION

Antimicrobial resistance is a global threat to human health and is associated with nearly 5 million annual deaths worldwide ^(1)^. Specifically, respiratory infections remain the deadliest transmissible diseases in the world, they are the leading cause of death among children younger than 5 years old, and the fourth overall leading cause of death globally ^(2, 3)^. For these reasons, respiratory infections caused by antibiotic-resistant bacteria are one of the greatest threats to human health. *Acinetobacter baumannii* is a multidrug-resistant pathogen that is primarily associated with nosocomial respiratory and bloodstream infections and a leading cause of global deaths attributable to antibiotic-resistance ^(1, 4)^. These facts have led the World Health Organization to identify drug-resistant *A. baumannii* as a critical pathogen for which the development of new therapies is desperately needed ^(5)^. Complicating the development of new therapeutic strategies to treat drug-resistant *A. baumannii* infections is the fact that this organism lacks conserved conventional toxins that could serve as therapeutic targets ^(6)^. Instead, *A. baumannii* has evolved a “persist and resist” virulence strategy ^(7)^ where multiple molecular factors each contribute to the ability of the bacterium to maintain cellular viability and proliferate within the host to cause infection ^(8)^.

Mammals have evolved numerous defenses to prevent the colonization and proliferation of pathogenic organisms. One key method exploits the metabolic requirements of invading microbes in a process collectively known as nutritional immunity. In response to infection, the mammalian host induces several systems to starve pathogens of essential transition nutrient metals, primarily iron (Fe), zinc (Zn), and manganese (Mn) ^(9)^. The ability of the vertebrate host to withhold nutrient metals from invading microbes is crucial to the defense against infection by bacterial pathogens ^(9)^. Specifically, host-mediated Fe starvation serves an important role in the restriction of microbial proliferation during infection. This is exemplified by the fact that elevated circulating Fe levels result in increased bacterial burdens in the lungs during pneumonia, enhanced dissemination from the lungs, and amplified overall mortality due to infectious disease ^(10–12)^. To counter this, bacterial pathogens have evolved a myriad of acquisition and sparing mechanisms to obtain essential metals and maintain cellular viability in the nutrient-limited environment of the host. The battle for Fe at the host-*A. baumannii* interface is a key determinate of infection outcome. The immune protein complex calprotectin, an important contributor to nutritional immunity, is released in the lungs during *A. baumannii* pneumonia, where it sequesters metals, including Fe and Zn, from the environment. Mice lacking calprotectin have increased *A. baumannii* burdens in their lungs during pneumonia ^(13)^. Additionally, *A. baumannii* strains with a limited capacity to acquire environmental Fe are attenuated for colonization of the host ^(14–16)^. Together, these findings support the idea that targeting systems that maintain bacterial fitness in the face of nutritional immunity is an attractive avenue for the development of new anti-*A. baumannii* therapeutics.

A well-characterized factor that contributes to *A. baumannii* virulence is the ability to attach to surfaces and develop mature biofilm structures ^(17)^. This virulence phenotype is conserved amongst clinical isolates of *A. baumannii* and aids in antimicrobial resistance, persistence on hospital surfaces, and attachment to host cells ^(18–20)^. A primary component of *A. baumannii* biofilm production is the assembly of ancient chaperone usher Csu pili. *A. baumannii* cells produce a radial coat of Csu pili that facilitate attachment to both abiotic and biotic surfaces. Following initial surface attachment, extensive networks of Csu pilin interactions form sheath-like structures that mediate cell to cell contacts, ultimately enabling mature biofilm formation ^(21)^. Csu-mediated biofilm production enhances *A. baumannii* survival on hospital surfaces, virulence, and antibiotic-resistance ^(20)^, therefore anti-biofilm therapeutics have been suggested as possible treatments against *A. baumannii* ^(21, 22)^. Expression of genes responsible for Csu pili production are regulated by both intra- and extracellular signals including nutrient metal availability, nucleotide secondary messengers, antibiotics, and quorum sensing ^(23–27)^. Understanding the regulatory mechanisms driving cellular surface attachment and biofilm formation is vital for developing effective *A. baumannii* control strategies.

The mechanisms that *A. baumannii* utilizes to maintain cellular viability to persist within the metal-starved environment of the host are incompletely understood. We have identified a genetic locus that contains several genes that function in the *A. baumannii* response to nutritional immunity and pathogenesis ^(13, 28, 29)^. Contained within this gene cluster is *ACX60_00365*, which is highly conserved amongst pathogenic *Acinetobacter* species and encodes for a previously uncharacterized membrane-bound acyltransferase belonging to the AT3 superfamily that we have named AimS ^(30)^. Several reports have indicated that *aimS* is regulated in response to nutrient metal limitation ^(31–33)^, and is induced within the murine lung during pneumonia, a metal-restricted environment ^(34)^. In this work we discovered that AimS contributes to bacterial fitness in low metal environments, including during pneumonic infection, through modulation of bacterial metabolism and maintenance of cellular energetics. Mechanistic studies revealed that AimS controls ATP content by reduction of Csu pilin production and biofilm formation. This occurs through repression of a previously uncharacterized TetR family transcriptional regulator that we have named CsuR. Collectively, these studies identified new layers of *A. baumannii* virulence phenotype regulation and uncovered a previously unappreciated sparing mechanism that bacteria employ in response to nutritional immunity through regulation of attachment factor production to maintain cellular energetics and pathogenesis.

## RESULTS

### *ACX60_00365* is induced by iron and zinc limitation and increases bacterial fitness in metal restricted environments

**Figure 1:**
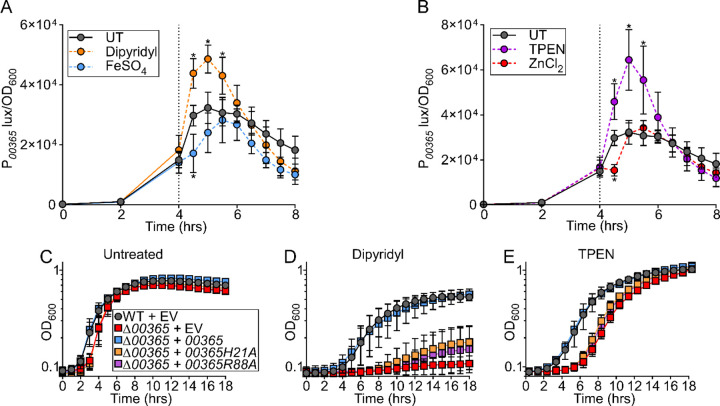
AimS mediates survival in low metal conditions (**A-B**) WT *A. baumannii* harboring a P_*00365*_-lux transcriptional fusion reporter plasmid was cultured to mid-log phase before being left untreated (UT) or treated with the indicated chelators or excess metals at 4 hrs (dotted line), transcriptional activity was monitored overtime by measuring luminescence every hour and dividing by the OD_600_ value of the culture. Data represent mean ± SD of at least 5 biological replicates performed technical triplicate. * *p* < 0.01, relative to UT, determined by Multiple Mann-Whitney tests. (**C-E**) WT or Δ*00365 A. baumannii* harboring either an empty vector (EV) control plasmid or a plasmid encoding the indicated *00365* alleles were cultured in LB medium alone (**C**) or with the addition of 200 μM of the iron chelator 2,2’-dipyridyl (**D**), or 50 μM of the zinc chelator TPEN (**E**), and bacterial fitness was monitored by recording OD_600_ of the culture every hour. Data represent mean ± SD of at least 6 biological replicates performed in technical triplicate.

The *A. baumannii* genome harbors a locus containing several genes that are induced by metal starvation and contribute to bacterial fitness in metal-restricted environments ^(13, 28, 29)^. Contained within this gene cluster is the uncharacterized gene *ACX60_00365* (*00365*). Several previous reports have indicated that *00365* is regulated in response to nutrient metal limitation ^(31–33)^, and is induced during pneumonic infection ^(34)^, suggesting that 00365 may function in the cellular response to metal starvation encountered within the host. To test this hypothesis, *00365* expression in response to metal restriction was evaluated using a luciferase-based transcriptional reporter construct expressed in WT ATCC 17978VU *A. baumannii*. Consistent with previous reports, ^(31, 33)^ *00365* was rapidly induced in response to either Fe or Zn limitation following treatment with the Fe or Zn chelators 2,2’-dipyridyl (dipyridyl) or N,N,Nʹ,Nʹ-tetrakis-(2-Pyridylmethyl)ethylenediamine (TPEN), respectively, and was repressed in response to excess metal treatment (Fig. 1A&B). To determine if 00365 participates in *A. baumannii* fitness in low metal environments, a Δ*00365* mutant strain was generated and assayed for fitness during Fe and Zn starvation by measuring bacterial growth over time. Compared to the WT strain, Δ*00365* showed decreased fitness when Fe or Zn were limiting, and the expression of *00365 in trans* in the Δ*00365* background completely restored these growth defects back to WT levels (Figs. 1C–E), indicating that the observed phenotypes were specifically due to the loss of *00365*.

*00365* encodes for a predicted acyltransferase belonging to the AT3 superfamily. Recent analysis of characterized AT3 family proteins revealed four residues that are 100% conserved within all AT3 proteins analyzed ^(35)^. These conserved residues include a histidine positioned within the first transmembrane helix. Additionally, AT3 proteins also contain a highly conserved RxxR domain positioned within the third transmembrane helix. Structural modeling has suggested that that these conserved protein features interact with acetyl coezyme-A to mediate acetyltransferase function, ^(35, 36)^ and mutational studies have indicated they are essential for the function of several bacterial AT3 proteins ^(35, 37, 38)^. These residues are conserved in 00365, therefore, their contribution to 00365 function during metal starvation was determined. While *in trans* expression of WT *00365* resulted in recovery of the Δ*00365* growth defect in both Fe and Zn-starved conditions, expression of *00365* alleles with a mutated conserved histidine (H21A) or with a perturbed RxxR motif (R88A) ablated complementation of both phenotypes (Figs. 1D&E). Collectively, these results indicate that *00365* encodes for an AT3 acyltransferase that is induced in response to metal starvation and functions to maintain bacterial fitness when either Fe or Zn is limiting. These findings motivated the naming of 00365 as AimS for acyltransferase induced by metal starvation.

### AimS contributes to the ability of *A. baumannii* to overcome nutritional immunity

**Figure 2:**
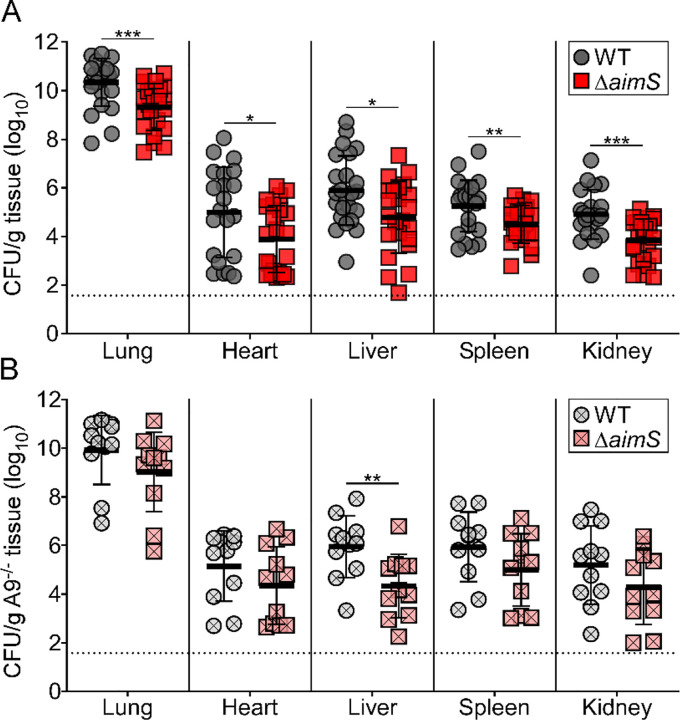
AimS contributes to *A. baumannii* defense against nutritional immunity. (**A-B**) Colony forming units (CFU) recovered from the indicated tissues of C57BL/6 (**A**) and calprotectin-deficient *S100A9*^*−/−*^ (**B**) C57BL/6 mice following intranasal infection with WT and Δ*aimS* at 36 hours post infection. Mean ± SD are shown. Each dot represents an individual organ. * *p* < 0.05, ** *p* < 0.01, *** *p* < 0.001 determined by Student’s t-test.

Nutritional immunity plays a critical role in the control of *A. baumannii* infection severity ^(15, 39, 40)^. This fact, combined with the finding that AimS functions in maintenance of *A. baumannii* fitness in metal-restricted conditions, led to the hypothesis that AimS also participates in *A. baumannii* infection. Utilizing a mouse model of pneumonic infection, C57BL6 mice were separately infected with either WT or Δ*aimS*. To assay for disease severity, bacterial burdens in the lungs, hearts, livers, spleens, and kidneys were enumerated at 36 hours post infection. Mice infected with the Δ*aimS* strain had ~ten-fold reduction in bacterial burdens in all organs collected, compared to mice infected with the WT strain (Fig. 2A), indicating that AimS increases *A. baumannii* colonization of the murine lung and dissemination to distal tissues during pneumonia. To test if the AimS-dependent increase in bacterial burdens during pneumonic infection is due to host-mediated metal sequestration, infection studies were repeated in *S100a9* ^*−/−*^ mice, which lack production of calprotectin ^(41)^. Calprotectin-deficient mice showed comparable bacterial burdens between mice infected with WT or Δ*aimS* in all organs tested, excluding the liver (Fig. 2B). This suggests that the reduced bacterial burdens within the organs of C57BL6 mice infected with Δ*aimS* were primarily due to nutritional-immunity mediated metal sequestration within the host, however additional factors appear to contribute to the defect in Δ*aimS* colonization in the livers of infected mice.

### AimS impacts pathogenesis independently of cellular metal content

To determine the mechanism by which AimS contributes to *A. baumannii* survival in low metal environments cellular metal content was quantified. The loss of *aimS* did not result in differences in cellular Fe or Zn concentrations, compared to WT cells, regardless of the addition of metal chelators (Fig. S1A&B). Moreover, the loss of *aimS* also did not impact cellular metal limitation responses in either metal replete or deplete environments, as measured using transcriptional fusion reporters that are induced by bacterial metal starvation responses ^(15, 33)^ (Fig. S1C&D). Taken together, these data suggest that AimS increases fitness during metal starvation via a mechanism that does not impact cellular metal content. As AT3 proteins often confer resistance to lysozyme through acylation of peptidoglycan ^(42)^, the contribution of AimS to lysozyme susceptibility was tested. No difference in the susceptibility to lysozyme-mediated killing between WT and the Δ*aimS* mutant was observed (Fig. S1E). Further, the expression of codon-optimized AT3 proteins previously characterized in other Gram-negative organisms were unable to complement the loss of *aimS* in low Fe conditions (Figs. S1F&G). Collectively, these results indicate that AimS contributes to the *A. baumannii* response to nutritional immunity via a mechanism that is independent of control of cellular metal content and appears to serve a function distinct from other characterized Gram-negative AT3 proteins.

### AimS is an *A. baumannii* metabolic regulator

**Figure 3:**
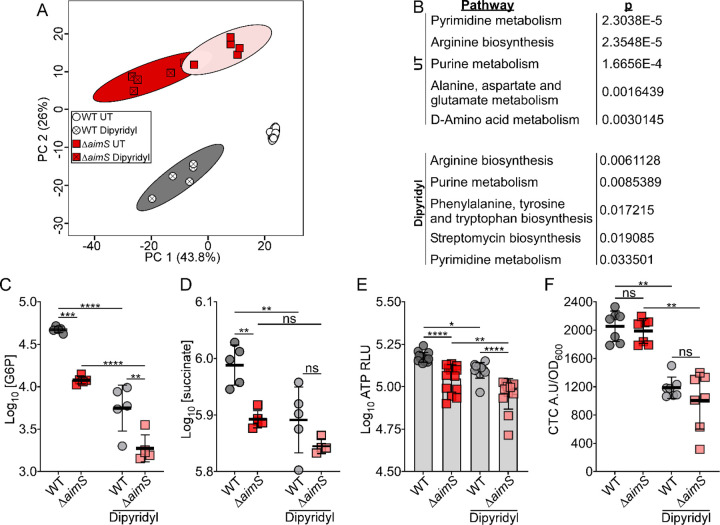
AimS modulates *A. baumannii* metabolism. (**A**) PCA plot of whole cell untargeted metabolomics of WT and Δ*aimS* cultured to mid-log phase in untreated (UT) conditions or Fe limited conditions (Dipyridyl). Each dot represents an individual biological replicate. (**B**) Top 5 altered metabolic pathways between WT and Δ*aimS* under Fe replete and deplete conditions. (**C & D**) Cellular concentration of glucose-6-phosphate (G6P) (**C**) and succinate (**D**) in WT and Δ*aimS* from untargeted metabolomics in (**A**). Mean ± SD are shown. Each dot represents an individual biological replicate. ** *p* < 0.01, ***, *p* < 0.001, **** *p* < 0.0001 determined by Šídák's multiple comparisons test. (**E**) Total cellular ATP concentration in mid-log phase cultures of WT and Δ*aimS* either left untreated or treated for 1 hr with 100 μM dipyridyl. Mean ± SD are shown. Each dot represents an individual biological replicate measured in technical triplicate. * *p* < 0.05, ** *p* < 0.01, **** *p* < 0.0001 determined by Tukey's multiple comparisons test. (**F**) Fluorescence of WT and Δ*aimS* strains after 20 minutes cultured in the presence of 5-cyano-2,3-ditolyl tetrazolium chloride in mid-log phase cultures of WT and Δ*aimS* cultured in Fe-replete and Fe-deplete (Dipyridyl) conditions. Mean ± SD are shown. Each dot represents an individual biological replicate measured in technical triplicate. ** *p* < 0.01 determined by Dunn's multiple comparisons test.

Recent reports have linked bacterial AT3 proteins to cellular metabolism and energy production ^(43, 44)^. These findings, coupled with the importance of Fe and Zn in several essential *A. baumannii* metabolic pathways ^(45, 46)^, led to the hypothesis that AimS may modulate *A. baumannii* metabolism. To explore this hypothesis, whole-cell untargeted metabolomic profiling was performed in the WT and Δ*aimS* strains under both Fe replete and deplete conditions. In these studies, the more pronounced low Fe phenotype was leveraged to study AimS function. Metabolic profiling observed large shifts in the cellular metabolome in both strains due to Fe limitation, while the WT and Δ*aimS* metabolomes were distinct from one another in both conditions tested (Fig. 3A). Pathway analysis revealed that several linked pathways including pyrimidine metabolism, purine metabolism, and arginine biosynthesis were significantly altered due to the loss of *aimS* in both conditions tested (Fig. 3B). One of the metabolites with the most dramatic differences in abundance between WT and Δ*aimS* was glucose-6-phosphate (G6P), a primary intermediate in central carbon metabolism ^(45)^. Fe limitation resulted in a reduction of G6P concentrations in both strains relative to Fe-replete conditions (Fig. 3C), and the loss of *aimS* resulted in a 3.9- and 3.1-fold reduction in G6P concentration relative to WT cells, in Fe replete and deplete conditions, respectively. Additionally, a reduction in the cellular concentration of the TCA cycle intermediate succinate was observed in the Δ*aimS* background (Fig. 3D). These data suggested a possible role for AimS in bacterial energetics. To test this, total cellular ATP concentrations were quantified in WT and Δ*aimS* cells. Consistent with metabolic profiling, Fe limitation reduced ATP concentrations in both strains, and the loss of AimS function resulted in the reduction of cellular ATP content in both conditions tested (Figs. 3E & S2). To determine if reduced ATP in Δ*aimS* is due to increased ATP utilization or decreased respiration, electron transport chain activity was assayed with the redox dye 5-Cyano-2,3-ditolyl tetrazolium chloride (CTC). Fe limitation resulted in a reduction of electron transport chain activity in both strains, illustrated by a decrease in the capacity of the cells to reduce the CTC dye (Fig. 3F). These findings, combined with the fact that cellular ATP pools are reduced in both strains during Fe starvation, indicate that the limitation of Fe results in a reduced cellular ability to produce ATP. However, no differences in CTC reduction were observed between the WT and Δ*aimS* cells in either condition tested, suggesting that the reduced ATP content in Δ*aimS* is likely due to increased utilization, as opposed to reduced production. Collectively these findings indicate that AimS functions in the regulation of metabolism and control of ATP consumption.

### Loss of an uncharacterized TetR type regulator encoded adjacent to the *csu* operon rescues AimS-dependent phenotypes

**Figure 4:**
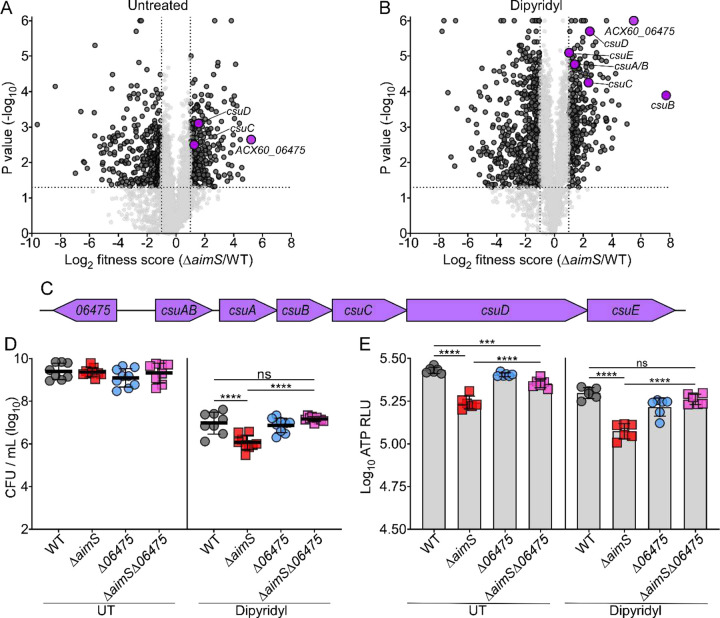
Loss of uncharacterized regulator encoded by *ACX60_06475* suppresses AimS-dependent phenotypes. (**A-B**) Fitness values of mutants harboring transposon insertions in the Δ*aimS* background relative to the WT background cultured in untreated (**A**) and low Fe (Dipyridyl) (**B**) conditions. Each dot represents a single gene within the *A. baumannii* genome. Vertical dotted lines denote fitness scores of −2 and 2, horizontal dotted line denote *P* value of 0.05. Genes that do not reach the statistical cutoff of *P* = 0.05 and fold change cut off of +/− 2 are grey. Genes encoding for production of the Csu pilus and adjacent TetR regulator *ACX60_06475* are highlighted in purple. (**C**) Cartoon representation of the *A. baumannii* genomic locus that encodes for the genes responsible for production of the Csu pilus. (**D**) CFU / mL of cultures of indicated *A. baumannii* strains cultured to mid-log phase in the untreated (UT) or low Fe (Dipyridyl) conditions. Mean ± SD are shown. Each dot represents an individual biological replicate. **** *p* < 0.0001 determined by Tukey's multiple comparisons test. (**E**) Total cellular ATP concentration in mid-log phase cultures of indicated *A. baumannii* strains cultured in Fe-replete (UT) and Fe-deplete (Dipyridyl) conditions. Mean ± SD are shown. Each dot represents an individual biological replicate measured in technical triplicate. *** *p* < 0.001, **** *p* < 0.0001 determined by Tukey's multiple comparisons test.

To explore the mechanism by which AimS impacts bacterial ATP utilization and fitness during Fe starvation, a transposon-sequencing (Tn-seq) screen was employed to identify genes that when disrupted conferred a fitness advantage to the Δ*aimS* mutant. High-density transposon mutant libraries consisting of ~100,000 individual insertion mutants were produced in both the WT and Δ*aimS* backgrounds. Transposon libraries were cultured under either Fe replete or deplete conditions before transposon junctions were amplified and sequenced. The resulting sequencing reads were then mapped to the *A. baumannii* 17978VU genome. These studies revealed that transposon insertions into several genes encoding for production of the ancient chaperone-usher Csu pili were highly selected for in the Δ*aimS* background compared to WT in both Fe replete and Fe deplete conditions (Figs. 4A&B). Additionally, strains maintaining transposon insertions into *ACX60_06475* (*06475*), which encodes for an uncharacterized TetR-type transcriptional regulator directly upstream of the *csu* operon (Fig. 4C), were also highly enriched in the Δ*aimS* mutant in both conditions.

To explore the role of 06475 in AimS-mediated reduced fitness, deletion mutants of *06475* were produced in both the WT and Δ*aimS* backgrounds and assayed for fitness during Fe starvation. The loss of *06475* alone did not impact growth in either condition, however, consistent with Tn-seq results, the loss of *06475* in the Δ*aimS* mutant background was sufficient to suppress the AimS-dependent growth repression back to WT levels (Fig. 4D). Additionally, the loss of *06475* was also sufficient to recover cellular ATP concentrations observed in Δ*aimS* back to WT levels in both Fe replete and deplete conditions (Fig. 4E). Collectively, these findings indicate that AimS impacts cellular ATP pools and fitness during metal limitation through a mechanism that is dependent on the 06475 transcriptional regulator.

### Transcriptional regulator ACX60_06475 induces *csu* gene expression in the Δ*aimS* background

**Figure 5:**
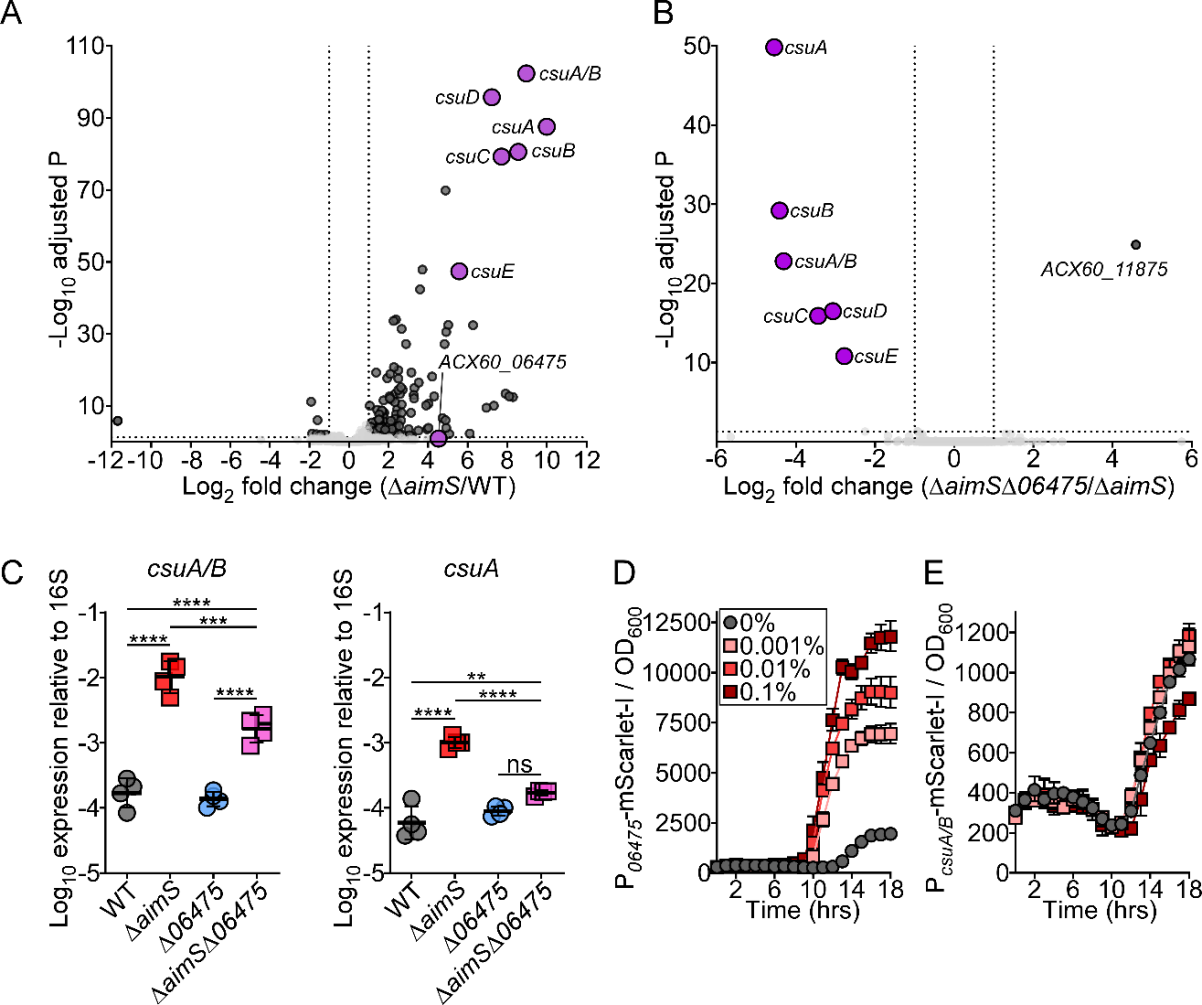
AimS induces biofilm production through modulation of CsuR transcriptional activity. (**A-B**) Comparisons of mid-log phase transcriptomes of (**A**) Δ*aimS* relative to WT and (**B**) Δ*aimS*Δ*06475* relative to Δ*aimS*. Each dot represents a single *A. baumannii* transcript. Vertical dotted lines denote fold changes of −2 and 2, horizontal dotted lines denote an adjusted *P* value of 0.05. Transcripts that do not reach the statistical cutoff of *P* = 0.05 and fold change cut off of +/− 2 are grey. Transcripts encoding for production of the Csu pilus and adjacent TetR regulator are highlighted in purple. (**C**) qRT-PCR analysis of *csuA/B* and *csuA* transcript abundance in the indicated *A. baumannii* strains cultured to mid-log phase in LB medium relative to 16S rRNA internal control. Mean ± SD are shown. Each dot represents an individual biological replicate measured in technical triplicate. ** *p* < 0.01, ***, *p* < 0.001, **** *p* < 0.0001 determined by Tukey's multiple comparisons test. (**D-E**) *E. coli* DH5α harboring a pBAD33.1-*06475* expression plasmid and either a P_*csuA/B*_-mScarlet-I (**E**) or P_*06475*_-mScarlet-I (**D**) transcriptional fusion reporter plasmid were cultured in the presence of the indicated L-arabinose concentrations in LB medium and transcriptional activity was monitored overtime by measuring mScarlet-I fluorescence every hour and dividing by the OD_600_ value of the culture. Data represent mean ± SD of 3 biological replicates performed in technical triplicate.

Although the deletion of *06475* was sufficient to suppress the AimS-dependent reduced fitness in low Fe environments and diminished cellular ATP content, the loss of *06475* did not impact cellular metal content (Fig. S3). This finding suggests that the loss of *06475* suppresses AimS-dependent phenotypes through transcriptional changes that do not impact the cellular capacity to acquire metals. To define these regulatory changes, transcriptomic profiling was performed on the WT, Δ*aimS*, and Δ*aimS*Δ*06475* strains. These studies revealed that the loss of *aimS* resulted in several large transcriptional changes, compared to WT cells. Consistent with the Tn-seq results, the transcripts with the highest differential abundance in Δ*aimS* cells compared to WT cells were within the *csu* genetic locus, including *06475,* although *06475* did not reach the threshold of statistical significance (Fig. 5A). The loss of *06475* in the Δ*aimS* background resulted in the statistically significant differential abundance of only seven transcripts, including repression of the entire *csu* operon (Fig. 5B), suggesting that *csu* gene expression is induced in an 06475-dependent manner in the Δ*aimS* strain. qRT-PCR corroborated these findings, as *csuA/B* and *csuA*, the two genes that maintain promoters within the *csu* locus, were both highly induced in the Δ*aimS* mutant, and the loss of *06475* resulted in repression of these genes back to near WT levels (Fig. 5C). To determine if induction of *csu* gene expression was due to increased promoter activation, a *csuA/B* transcriptional fusion plasmid was created. In agreement with qRT-PCR findings, induction of *csuA/B* correlated with increased *06475-*dependent promoter activity in the Δ*aimS* background (Fig. S4A). These studies also revealed that the loss of *06475* suppressed *csuA/B* expression in WT cells when cultures entered stationary phase. Taken together, these findings suggest that 06475 may impact *csu* gene regulation through binding of the *csuA/B* promoter. This hypothesis was tested using a two-plasmid system independently expressing *06475* and P_*06475*_ or P_*csuA/B*_ transcriptional fusion reporters in a non-heterologous *E. coli* host (Fig. S4B). Expression of 06475 resulted in a dose-dependent induction of *06475* promoter activity (Fig. 5D). However, 06475 expression alone was not sufficient to alter *csuA/B* promoter activity in *E. coli* (Fig. 5E), indicating that additional factors absent in *E. coli* likely contribute to 06475-mediated increased *csuA/B* promoter activation in the Δ*aimS* background. Collectively, these studies indicate that 06475 regulates *06475* and *csuA/B* expression through modulation of promoter activity within the intergenic region between *06475* and *csuA/B*. Based on these findings, we named 06475 as CsuR for Csu pilin regulator.

### Overproduction of Csu pili is energetically costly and reduces fitness when Fe is limiting

**Figure 6:**
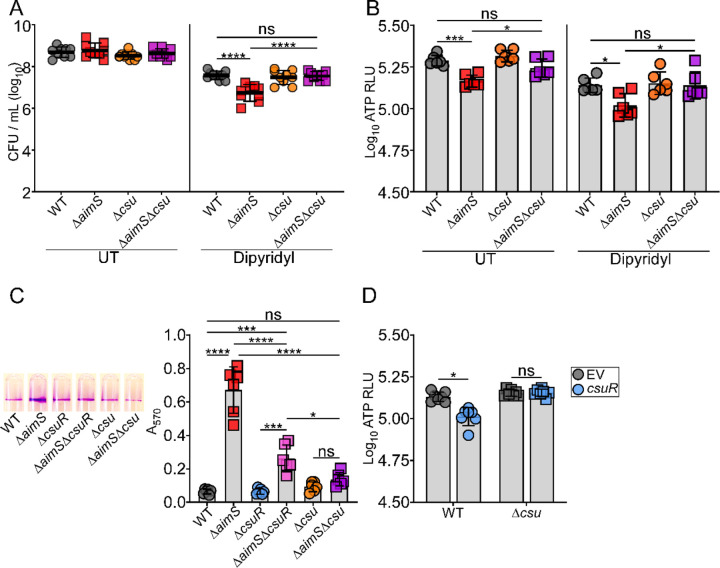
AimS represses Csu pilin production to overcome nutritional immunity. (**A**) CFU / mL of cultures of indicated *A. baumannii* strains cultured to mid-log phase in the untreated (UT) or low Fe (Dipyridyl) conditions. Mean ± SD are shown. Each dot represents an individual biological replicate. **** *p* < 0.0001 determined by Tukey's multiple comparisons test. (**B**) Total cellular ATP concentration in mid-log phase cultures of indicated *A. baumannii* strains cultured in Fe-replete (UT) and Fe-deplete (Dipyridyl) conditions. Mean ± SD are shown. Each dot represents an individual biological replicate measured in technical triplicate. * *p* < 0.05, *** *p* < 0.001, determined by Tukey's multiple comparisons test. (**C**) Static biofilm formation on polystyrene of indicated *A. baumannii* strains measured via crystal violet staining at 16 hrs of incubation. Mean ± SD are shown. Each dot represents an individual biological replicate measured in technical triplicate. * *p* < 0.05, ** *p* < 0.01, *** *p* < 0.001 determined by Dunn's multiple comparisons test. (**D**) Total cellular ATP concentration in mid-log phase cultures of indicated *A. baumannii* strains harboring either an empty vector plasmid (EV) or a *csuR* overexpression plasmid (*csuR*). Mean ± SD are shown. Each dot represents an individual biological replicate measured in technical triplicate. * p < 0.001 determined by unpaired t test with Welch correction.

The above results suggest that *A. baumannii* utilizes AimS activity to repress CsuR-mediated induction of *csu* gene expression, resulting in maintenance of cellular ATP pools, particularly in low Fe environments where ATP production is limited. To test this hypothesis Δ*csu* mutants were generated in both the WT and Δ*aimS* backgrounds by deletion of the *csu* operon (*csuA/B,A,B,C,D,* and *E*) and fitness in Fe replete and deplete conditions was tested. While the loss of the *csu* operon alone did not impact growth in either condition tested, the loss of *csu* genes was sufficient to suppress the AimS-dependent reduced fitness in a low Fe environment, consistent with transcriptional data (Fig. 6A). Additionally, the loss of the *csu* operon in the Δ*aimS* mutant was also sufficient to restore cellular ATP content back to WT levels in both Fe replete and deplete conditions (Fig. 6B). These findings collectively indicate that CsuR-mediated induction of *csu* gene expression is responsible for the observed reduction in cellular ATP content and loss of fitness in the Δ*aimS* mutant when Fe is limiting. These findings establish causal linkage between CsuR-mediated induction of *csu* genes and reduced fitness during Fe limitation in the absence of AimS.

*A. baumannii* Csu pili mediate attachment to biotic and abiotic surfaces and biofilm development ^(19, 20, 47)^, suggesting that CsuR-mediated induction of *csu* gene expression in the absence of AimS would result in an increase in biofilm formation. Indeed, the loss of AimS activity resulted in increased biofilm production (Fig. S5). This phenotype appeared to be dependent on CsuR-mediated induction of *csu* gene expression as the loss of CsuR or Csu pili were sufficient to ablate the phenotype (Fig. 6C). Together, these findings suggest that CsuR-dependent regulation of *csu* gene expression modulates biofilm production and cellular energetics. Consistent with this hypothesis, overexpression of *csuR* resulted in a Csu pilin-dependent reduction of cellular ATP content (Fig. 6D). Collectively, these results indicate that *A. baumannii* utilizes AimS to control surface attachment, biofilm production, and cellular ATP pools through modulation of CsuR transcriptional activity.

## DISCUSSION

To prevent pathogen proliferation the vertebrate host leverages the nutritional requirements of invading organisms in a process known as nutritional immunity. Specifically, induction of mechanisms that starve microbes of essential iron (Fe) is a significant contributor to the mammalian immune response. Therefore, the ability to survive within Fe-limited environments is a key virulence determinate of human pathogens ^(9)^. Several previous reports have established that the ability to overcome host-imposed Fe limitation contributes to the pathogenesis of the extensively drug-resistant Gram-negative bacterium *Acinetobacter baumannii* ^(14)^. However, there is an incomplete understanding of how *A. baumannii* overcomes host-imposed metal sequestration to maintain viability during infection. Here, we report a previously unknown mechanism that bacteria utilize to maintain fitness in the face of nutritional immunity through repression of genes encoding for pili production to maintain cellular energetics. This survival strategy is controlled by a previously uncharacterized acyltransferase that we have named AimS and a transcriptional regulator that we have named CsuR.

In response to nutritional immunity, bacterial cells alter several pathogenic phenotypes. Specifically, Fe limitation leads to the induction of biofilm production in a wide range of organisms, including *A. baumannii* ^(48)^. *A. baumannii* biofilm production increases bacterial pathogenesis by mediating attachment to both hospital surfaces and host cells, and by increasing resistance to antibiotics ^(20, 49–51)^. These findings have led to the suggestion of developing antibiofilm agents as therapeutics against drug-resistant *A. baumannii* ^(21, 52)^. The production of extracellular structures, including components required for biofilm production, are energetically costly ^(53, 54)^. We observed that Fe starvation results in a reduction of flux through the electron transport chain, and diminished ATP concentrations in *A. baumannii*, which is consistent with previous reports in several different bacterial species ^(55–58)^. Additionally, we previously reported that *A. baumannii* reduces the abundance of several components of the electron transport chain when exposed to the metal chelating immune protein complex calprotectin ^(31)^. Taken together, these findings suggest that during persistence within the Fe-starved environment of the host, *A. baumannii* has a reduced capacity to produce ATP. Here, we discovered that the production of Csu pili appears to be an energy-intensive process. Csu pili are large surface-anchored attachment factors that radially coat the surface of *A. baumannii* cells. Individual pili can consist of thousands of pilin subunits ^(59)^ and exceed the length of a bacterial cell by several times ^(21)^. To facilitate pilin production, each synthesized protein subunit must be mobilized across the bacterial inner-membrane in an ATP-dependent manner ^(60)^. Once produced, Csu pili bind one another to form extensive sheath-like network structures that anchor the *A. baumannii* community, enabling biofilm formation ^(21)^. This creates a fundamental energetic conflict for the cell during infection, which must synthesize energetically costly biofilm structures at a time when ATP production is reduced. Collectively, these results support a model where in Fe-starved environments, like those encountered during host infection, *A. baumannii* utilizes AimS as a sparing response to modulate the production of Csu pili. This response enables the cell to balance the high energy cost of producing attachment structures with the diminished capacity to generate ATP imposed by nutritional immunity. This regulatory strategy likely represents a broader class of energy-sparing adaptations that enable bacterial persistence under host-imposed nutritional stress.

The promoter of the *csu* operon, as well as the divergently encoded *csuR* promoter, both contain putative binding sites for HTH transcriptional regulators ^(20)^. This, coupled with the fact that TetR type regulators are often encoded alongside the operon they target for regulation, resulted in speculation that CsuR may directly regulate its own expression and expression of *csu* genes ^(20, 30)^. However, this hypothesis has not been explicitly explored in the literature. Here, we discovered that CsuR induces expression of both *csuR* and *csuA/B* through increased promoter activity, confirming these previous hypotheses and characterizing a new regulator of *csu* gene expression. CsuR is a TetR-type transcriptional regulator, which typically function as transcriptional repressors. However, several reports have shown that, like CsuR, other TetR-type regulators function as direct transcriptional activators of target gene expression ^(61–65)^. Furthermore, this work has uncovered an additional layer of regulation in this pathway where AimS represses the activity of CsuR, resulting in repression of *csu* gene expression. The mechanism by which AimS modulates CsuR activity remains unknown, however TetR family regulators are typically one-component signal transduction proteins that bind a cognate ligand, ultimately dictating their regulatory activity ^(66)^. This, coupled with our finding that AimS modulates *A. baumannii* metabolism suggests that metabolic changes due to the loss of *aimS* may result in the observed impact on CsuR activity. Previous reports have found that treatment with trimethoprim, a drug that inhibits folate metabolism and thus nucleotide synthesis, modulates *csu* gene expression ^(67)^. Additionally, several mutations in folate biosynthesis, nucleotide secondary messenger production, and nucleotide production result in altered biofilm formation ^(68, 69)^. These facts, coupled with our finding that the metabolic pathways most impacted by the loss of AimS are production of nucleotides, suggest a possibility that changes in nucleotide metabolism are responsible for CsuR-mediated induction of *csu* gene expression. Additionally, the loss of *csuR* was not sufficient to completely return *csu* gene expression back to WT levels in the Δ*aimS* background, indicating that additional factors contribute to induction of *csu* expression in the absence of *aimS*.

The gene encoding for *aimS* along with the divergently-encoded histidine utilization genes, which also contribute to *A. baumannii* pathogenesis ^(29)^, make up an evolutionarily stable gene cluster that is conserved across the *Acinetobacter* species, with high conservation in pathogenic *Acinetobacter* species ^(30)^. The locus containing *csu* genes, including *csuR,* also compose an evolutionarily stable gene cluster that is highly conserved within the pathogenic strains of *Acinetobacter,* but is rare in non-pathogenic strains ^(30)^. In *A. baumannii* clinical isolates the *csu* genomic region has the highest variability in expression of any region within the genome ^(70)^. Further, a study of over 1,000 *A. baumannii* genomes found that the *csu* locus is the location of dozens of independent IS element insertions by 13 different genomic elements ^(71)^, and several additional mutations within this locus have been recorded in both lab-adapted and clinical isolates resulting in both increased and decreased expression ^(70, 72)^. Consist with these observations, a high level of heterogeneity in biofilm formation is observed in *A. baumannii* clinical isolates ^(18, 73)^. Collectively these studies indicate that this highly conserved region of the *A. baumannii* genome is under tremendous selective pressure in the clinic. This hypothesis is supported by the fact that the *csu* cluster has been independently lost multiple times in circulating clades of *A. baumannii* ^(30)^. This selection may be because stimulation of IL-8 production by SW480 cells correlates with the amount of CsuA/B produced by *A. baumannii* clinical isolates ^(20)^. These findings suggest that *A. baumannii* must finely tune the production of the Csu pili to balance the need for attachment and biofilm production while avoiding activation of the host immune system. This is supported by the fact that expression of the *csu* gene cluster is regulated by several cellular factors and regulatory signals stemming both intra- and extracellularly ^(74–78)^. In this report we found that *A. baumannii* utilizes AimS to repress *csu* gene expression through the regulator CsuR to conserve ATP content in low Fe environments, where ATP production is impaired. These findings may represent an additional selective pressure on this genomic region to maintain cellular energetics during Fe starvation; a stressor encountered during host colonization.

AT3 family acyltransferases are encoded for in all domains of life, although they are particularly prominent in bacterial species. Bacterial AT3 proteins are separated into two classes: those that are fused to an SGNH domain-containing C-termini that is essential for acyl transfer, and AT3 domain-alone proteins. In Gram-negative species AT3 proteins are imbedded within the inner membrane. AT3 domains form a pore to allow acyl molecules to traverse the cellular membrane to then be attached to extracellular target molecules ^(36, 79)^. Although AT3 alone proteins are far more common than SGNH-linked proteins, much less is known about their function, and the mechanism of acyl transfer by proteins that contain only AT3 domains remains unknown ^(80, 81)^. AimS encodes for an AT3 stand-alone protein. Characterized bacterial AT3 alone proteins most commonly function in the acylation of extracellular carbohydrate molecules, although recent reports have indicated that they also transfer acyl groups to small molecules, lipids, and proteins ^(43, 81)^. Here, we have uncovered previously unappreciated roles for AT3 family acyltransferases in the bacterial response to nutritional immunity through regulation of attachment factor expression and biofilm production. Although the acyltransferase activity of AimS is essential for all the observed phenotypes, the direct acylation target(s) of AimS, and how these acylation events result in repression of CsuR transcriptional activity and downstream modulation of biofilm production, remains unknown. Recent reports have indicated that *Klebsiella pneumoniae* and *Streptococcus pneumoniae* utilize AT3 family proteins to regulate cellular metabolism. Specifically, *S. pneumoniae* uses AT3-mediated peptidoglycan acetylation as an extracellular signal to regulate bacterial metabolism and growth, linking the acetylation state of surface carbohydrates to the metabolic status of the cell ^(44)^. Taken together, these findings suggest that utilization of AT3 proteins to alter cellular metabolic homeostasis may be a conserved mechanism that bacteria employ to respond to stress conditions.

## METHODS:

### Animal experiments:

Mouse experiments were performed using female 8–10-week-old C57BL/6J mice supplied by Jackson Laboratories. Animals were maintained at the Vanderbilt University Medical Center (VUMC) Animal Facilities, with a 12 hr light-dark cycle and food and water provided ad libitum. For experimental endpoints, animals were humanely euthanized. All sanimal experiments were approved and performed in compliance with the Institutional Animal Care and Use Committee (IACUC) of Vanderbilt University (protocol number M1900043–02) and conform to policies and guidelines established by VUMC, the Animal Welfare Act, the National Institutes of Health, and the American Veterinary Medical Association.

### Bacterial strains and growth conditions:

Bacterial strains used in this study are listed in Table S1. Bacteria were cultured in lysogeny broth (LB) or on LB with 1.5% w/v agar (LBA). For the purposes of plasmid maintenance and screening for mutational insertion, antibiotics were added at the following concentrations: carbenicillin, 75 μg/mL; chloramphenicol, 15 μg/mL; kanamycin, 40 μg/mL; tetracycline, 5 μg/mL (*E. coli*), and 10 μg/mL (*A. baumannii*).

### Cloning and genetic manipulation:

All plasmids and primers used in this study are listed in Tables S1 and S2, respectively. All plasmids in this study were constructed using HiFi Assembly ^(19)^ following the manufacturer’s protocol ^(19)^. Prior to use all cloned vectors were confirmed by Sanger or whole-plasmid sequencing. *P*_*aimS*_*, P*_*fbsB*_*, P*_*zigA*_-*lux* transcriptional fusion reporter plasmids were constructed by amplifying upstream regions of each gene containing the predicted promoters (phiSITE) using *A. baumannii* genomic DNA as a PCR template and cloned into SacI and BamHI digested pMU368(tet)-*lux* vector. *P*_*csuA/B*_ and *P*_*csuR*_-*mScarlet-I* transcriptional fusion reporters were constructed by first amplifying the sfGFP and mScarlet-I dual reporter cassette from pCG-VmS ^(82)^ and cloning into BamHI and HindIII digested pWH1266 using the melt and reanneal method ^(83)^ creating vector pWH1266-CG-VmS. The *csuA/B* and *csuR* promoters consisting of the whole intergenic regions between the two genes were amplified using *A. baumannii* genomic DNA as a PCR template and cloned into BamHI and HindIII digested pWH1266-CG-VmS.

For construction of the Δ*aimS* and Δ*csu*, deletion constructs 1,000 bp of DNA in both the 5′ and 3′ flanking regions surrounding the gene (*aimS*) or operon (*csuA/B,A,B,C,D,E*) were amplified using *A. baumannii* genomic DNA as a PCR template and cloned into XbaI and BamHI digested pFLp2 vector. For construction of the Δ*csuR* construct, 500 bp of DNA in both the 5′ and 3′ flanking regions surrounding gene were amplified using *A. baumannii* genomic DNA as a PCR template and the kanamycin resistance gene *aphA* was amplified by PCR from the vector pUCK1, these products were cloned into XbaI and BamHI digested pFLp2 vector. To produce indicated mutant strains gene deletion constructs were introduced into *A. baumannii* by tri-parental conjugation using an HB101 *E. coli* strain containing the helper plasmid pRK2013 on LBA for ~18 hrs. Matings were plated onto LBA containing carbenicillin 75 μg/mL and chloramphenicol 15 μg/mL to select for strains containing the integrated allelic exchange plasmid. Strains were then plated onto LBA containing 10% sucrose to select for clones that had resolved the integrated plasmid and resulting sucrose-resistant colonies. Resulting sucrose-resistant colonies were patched onto LBA supplemented with kanamycin 40 μg/mL. Deletion of target gene(s) was confirmed first by PCR with primers external to the deleted gene(s), followed by whole-genome sequencing (Seq-center & Plasmidsaurus).

The *aimS* complementation plasmid was constructed by amplifying *aimS* and the native upstream predicted promoter (phiSITE) using *A. baumannii* genomic DNA as a PCR template and cloned into SalI and BamHI digested pWH1266 vector. AimS H21A and R88A mutant allele expression constructs were generated by site-directed mutagenesis. The pWH1266-*aimS* vector was amplified in a PCR reaction with primers encoding the indicated point mutants. The resulting PCR product was DpnI digested and transformed into *E. coli*. Plasmids harboring the desired mutations were screened by Sanger sequencing. Constructs expressing AT3 only proteins from other Gram-negative bacteria were constructed as follows. The indicated genes were codon optimized to *Streptococcus pneumoniae* using the IDT Codon Optimization Tool (Table S3) and the resulting DNA sequences were synthesized into gblocks (IDT). AT3 alleles were PCR amplified using the resulting gblocks as templates and the *aimS* native promoter region was amplified using *A. baumannii* genomic DNA as a PCR template. The resulting fragments were cloned into SalI and BamHI digested pWH1266 vector.

For construction of the *csuR* overexpression construct, the *csuR* gene was amplified using *A. baumannii* genomic DNA as a PCR template and cloned into XhoI and KpnI digested pLDP29 vector, downstream of the strong constitutive *rpsA* promoter.

### Luciferase and mScarlet-I transcriptional reporter assays:

Overnight cultures of indicated strains harboring indicated transcriptional reporter plasmids were started from single colonies in LB broth with the addition of tetracycline (luciferase) or carbenicillin (mScarlet-I). The following day, overnight cultures were back diluted into fresh LB medium with antibiotics 1:50, and allowed to grow with shaking at 37 °C for 1 hr. Back diluted cultures were used to inoculate the wells of black clear bottom 96 well microtiter plates 1:100 with indicated media containing antibiotics. Measurement of mScarlet-I reporter activity were performed as previously reported ^(84)^. Plates were cultured with continuous shaking at 37 °C and total luciferase or fluorescence (ex: 575 nm, em: 635 nm) and OD_600_ were recorded every 60 min. Treatment to reporter strains were added at 4 hrs where indicated. Data are reported as luciferase or fluorescence divided by the OD_600_ of the culture.

### Bacterial growth curves:

Overnight cultures of indicated strains were started from single colonies in LB broth, with antibiotics when appropriate. The following day overnight cultures were back-diluted into fresh LB media 1:50, and allowed to grow with shaking at 37 °C for 1 hr. Back diluted cultures were used to inoculate the wells of 96 well microtiter plates 1:100 with indicated media. Plates were cultured with continuous shaking at 37 °C for 18 hrs and OD_600_ was recorded every 60 min.

### Mouse infections:

*A. baumannii* overnight cultures were diluted 1:1000 in fresh LB medium for 3.5 hrs when cultures were harvested, washed twice in sterile PBS, and suspended in sterile PBS to ~1×10^10^ CFU/mL and serially diluted and spot-plated to confirm equivalent bacterial concentrations between strains and experiments. Prior to infections, mice were anesthetized by intraperitoneal injection of 2,2,2 tribromoethanol diluted in sterile PBS. Anesthetized mice were infected intranasally with a 40 μL volume of the bacterial inoculum (2.4 × 10^8^ - 6.0 × 10^8^ CFU). Infections were allowed to proceed for 36 hrs, during which time mouse weight and survival was monitored. Mice were euthanized by forced CO_2_ inhalation followed by cervical dislocation, and lungs, hearts, livers, spleens, and kidneys were sterilely harvested. Organs were homogenized and serial dilutions of homogenized tissues were spot plated onto LB agar for enumeration.

### Inductively coupled plasma mass spectrometry:

Overnight cultures of the indicated *A. baumannii* strains were used to inoculate fresh 5 mL cultures of fresh LB medium in metal-free 15 mL conical tubes 1:1000. Strains were cultured for 4 hrs with shaking at 37 °C before being left untreated or treated with either 100 μM dipyridyl or 30 μM TPEN, these conditions did not impact growth of any strains tested, and cultured for an additional 1 hr. Bacterial cells were collected via centrifugation and washed twice with 1X sterile PBS. Bacterial pellets were digested in 200 μL 70% Optima-grade nitric acid at 65°C overnight, then diluted with UltraPure water to 20% nitric acid for analysis. Elemental quantification was conducted using an Agilent 7700 ICP-MS attached to an ASX-560 autosampler. The settings for analysis were cell entrance = −40 V, cell exit = −60 V, plate bias = −60 V, OctP bias = −18 V, and helium flow = 4.5 mL/min. Optimal voltages for extract 2, omega bias, omega lens, OctP RF, and deflect were empirically determined. Calibration curves for elements were generated using ARISTAR ICP standard mix. Samples were introduced by peristaltic pump with 0.5-mm-internal-diameter tubing through a MicroMist borosilicate glass nebulizer. They were initially taken up at 0.5 rps for 30 seconds, followed by 30 seconds at 0.1 rps to stabilize the signal. Spectrum mode analysis was performed at 0.1 rps, collecting three points across each peak and conducting three replicates of 100 sweeps for each element. The sampling probe and tubing were rinsed with 2% nitric acid for 30 seconds at 0.5 rps between each sample. Data were acquired and analyzed using Agilent MassHunter workstation software version A.01.02.

### Lysozyme killing assays:

Overnight cultures of WT and Δ*aimS* were used to inoculate fresh LB medium with a 1:100 dilution and cultured with shaking at 37 °C. At 5 hrs, cultures were collected, washed 1X in TBS and normalized to an OD_600_ of 0.3 in TBS, then diluted 1:100 in fresh TBS ± 1 mg/mL lysozyme. TBS suspended bacteria were incubated static at 37 °C. Bacterial CFU were enumerated via serial dilution and plating to LBA plates at the indicated time points.

### ATP production:

Overnight cultures of the indicated *A. baumannii* strain were used to inoculate LB medium with and without antibiotics where appropriate with a 1:1000 dilution and cultured with shaking at 37 °C. At 4 hrs cultures were left untreated, treated with 30 μM TPEN or 100 μM dipyridyl. These conditions did not impact growth of any strains tested. At 5 hrs the OD_600_ of cultures were recorded and 250 μL of the cultures were centrifuged in a tabletop centrifuge at max speed for 2 minutes and supernatants removed. Cultures were normalized to an OD_600_ of 0.5 in sterile 1X PBS. In technical triplicate 100 μL of normalized cultures were then mixed with an equal volume of Promega CellTiter-Glo^®^ 2.0 reaction mixture in the wells of a black 96 well plate, according to manufacturer’s protocol, and light production was recorded in a plate reader.

### CTC assay:

Respiration was measured using the redox dye 5-Cyano-2,3-ditolyl tetrazolium chloride (CTC) according to^(85)^ with minor alterations. Indicated *A. baumannii* strains were inoculated into fresh 5 mL of LB medium at a 1:1000 dilution from LB overnight cultures and cultured at 37 °C with shaking. At 4 hrs 1 mL of cultures were moved to 1.5 mL eppendorf tubes 37 °C and either left untreated or treated with 100 μM dipyridyl and returned to shaking at 37 °C for an additional hour. One hundred μL of 5 hr cultures were then mixed with 2 mM CTC dye dissolved in ethanol in the wells of a black microtiter plate and incubated at 37 °C with shaking for 20 mins. Finally, the OD_600_ values of the mixture and fluorescence were measured with an excitation of 485 nm and an emission of 630 nm. Data are reported as arbitrary fluorescence units (A.U) divided by the OD_600_ of the culture.

### Metabolomics:

Five biological replicates of WT and Δ*aimS* overnight cultures were used to inoculate 3 mL of fresh LB medium and cultured with shaking at 37 °C for 3 hrs. To overcome slower growth in the Δ*aimS* mutant strain ~1 million CFU of WT cultures and ~10 million CFU of Δ*aimS* cultures were used to separately inoculate 10 mL of fresh LB medium with and without the addition of 175 μM dipyridyl. Samples were then cultured for 6 hrs when 100 μL were collected and serially diluted and plated on LB medium to ensure relative CFU counts between strains and conditions. The remaining cells were collected via centrifugation and supernatants removed. Bacterial cell pellets were washed in 1.5 mL 50 mM ammonium formate and flash frozen in liquid nitrogen and stored at −80 °C.

Bacterial pellets stored at −80 °C were analyzed using liquid chromatography-high resolution tandem mass spectrometry (LC-HRMS and LC-HRMS/MS)-based metabolomics at the Vanderbilt Center for Innovative Technology (VCIT), employing previously described methods ^(86, 87)^. Briefly, pellets were thawed on ice and lysed in 500 μL ice-cold lysis buffer (1:1:2, acetonitrile: methanol: ammonium bicarbonate 0.1M, pH 8.0) followed by probe tip sonication with 10 pulses at 30% power. Protein content was determined by BCA assay following manufacturer’s protocol (Thermo Fisher Scientific). Two hundred μg total protein per sample was collected and adjusted to 200 μL total volume with lysate buffer. Isotopically labeled standards (phenylalanine and biotin) were spiked into each sample prior to processing to assess sample process variability. Deproteinization was performed by cold methanol precipitation via overnight incubation at −80 °C, followed by centrifugation at 4 °C for 10 mins. Cleared supernatant were transferred to a new vial and dried by cold vacuum centrifugation.

Prior to mass spectrometry analysis, individual hydrophilic extracts were reconstituted in 100 μL acetonitrile/water (80:20, v/v) containing isotopically labeled standards, tryptophan, inosine, valine, and carnitine. Samples were then centrifuged for 5 min at 10,000 rpm to remove insoluble material prior to analysis. A pooled quality control sample (pooled QC) was prepared by mixing equal volumes of individual samples following reconstitution. The pooled QC sample used for column conditioning (8 injections before sample analysis), for retention time alignment, to assess mass spectrometry instrument reproducibility, and to determine batch and individual sample acceptance. Samples were randomized and injected once. Quality assurance practices were applied to assess the analytical method performance, and a system suitability sample was analyzed to verify the stability of retention times, peak shapes, and peak areas before and after sample batch analysis. Global, untargeted mass spectrometry analyses were performed on a high-resolution Q-Exactive HF hybrid quadrupole-Orbitrap mass spectrometer (Thermo Fisher Scientific) equipped with a Vanquish UHPLC binary system (Thermo Fisher Scientific) using hydrophilic interaction liquid chromatography in negative ion mode (6 μL injections) using previously described chromatography and instrumentation methods ^(88, 89)^.

Data processing and statistical analysis on metabolomic data were performed as follows. Mass spectrometry raw data were imported, processed, normalized, and reviewed using Progenesis QI v.3.0 (Non-linear Dynamics). All MS and MS/MS sample runs were aligned against a pooled QC reference run. Unique ions (retention time and m/z pairs) were de-adducted and de-isotoped to generate unique “features” (retention time and m/z pairs). Data were normalized to all features and significance was assessed using p-values generated using ANOVA (analysis of variance) from normalized compound abundance data. Tentative and putative annotations were determined by using accurate mass measurements (< 10 ppm error), isotope distribution similarity, retention time and fragmentation spectrum matching (when applicable) ^(90)^ by searching the Human Metabolome Database (HMDB) ^(91)^ and the VCIT’s curated in-house reference library. Annotations (Confidence Level 1–3) were assigned for all compounds that matched any of the searched libraries or databases. Metaboanalyst 5.0 (www.metaboanalyst.ca/) was used to perform pathway and metabolite enrichment analyses from annotated compounds with statistical significance (p-value ≤ 0.05) ^(92)^.

### Transposon mutant library construction and transposon-sequencing:

*A. baumannii* high density transposon mutant libraries were prepared as previously described ^(93)^. The Himar1 transposon containing vector pJNW684 was introduced into both WT and Δ*aimS* mutant strains via mating with the *E. coli* mating strain SM10λpir. Prior to matings *A. baumannii* and strains SM10λpir harboring pJNW684 were cultured in LB medium overnight at 37 °C. Overnight cultures were washed three times in 1X PBS and SM10λpir+pJNW684 was mixed with recipient *A. baumannii* strains at a 2:1 ratio. Bacterial mating mixtures were plated onto LB agar plates and matings were allowed to proceed for 4 hrs at 37 °C, then resuspended from plates in 1X PBS and a fraction of the mating was spread across the surface a LB agar plates containing 40 μg/mL kanamycin and 15 μg/mL chloramphenicol. Library pools containing approximately 100,000 colonies in both genetic backgrounds were scraped from selection plates, glycerol was added to a final concentration of 20%, and transposon libraries were stored at −80 °C.

For transposon sequencing studies identical conditions used for untargeted metabolomics were utilized. Triplicate frozen aliquots of both the WT and Δ*aimS* mutant libraries were brought up in 10 mL of fresh LB media and cultured for 3 hrs with shaking at 37 °C. From these library cultures ~1 million CFU (≥10X library coverage) were used to individually inoculate fresh 10 mL cultures of LB medium alone and LB medium + 175 μM dipyridyl in biological triplicate. The resulting cultures were grown with shaking at 37 °C for 6 hrs (~10 bacterial generations). Bacterial cells were separated and supernatants discarded and gDNA was isolated using a DNeasy (Qiagen) kit according to the manufacturer’s protocol.

DNA libraries were prepared for sequencing using the homopolymer tail-mediated ligation PCR technique^(94)^. gDNA was sheared by sonication using the Covaris LE220 instrument to generate 350 bp fragments. Sheared DNA was treated with terminal deoxytransferase to generate a 3’ poly C-tail sequence, and two rounds of nested PCR were employed to amplify transposon junction regions. These products were multiplexed using 8-bp indexing primers listed in Table S3 and sequenced on the Illumina NextSeq at Tufts University Core Facility. Following sequencing, reads were trimmed, filtered for quality, and mapped to the ATCC *A. baumannii* 17978 genome (accession NZ_CP012004). A “Dval” score was assigned to each gene in each library pool, representing the aggregate number of reads for all transposon insertions within a gene in a given library sample, divided by the total number of predicted reads for that gene based on its size and the total number of reads obtained for the library pool. Essential genes in each genetic background were classified as genes that had an average transposon insertion count of less than 20 across all 3 replicates. Fitness scores for each non-essential gene were calculated by dividing the Dval for a given gene under the indicated condition in the Δ*aimS* library to the WT library. Fitness scores were Log_2_-transformed and an average Log_2_ fitness score was calculated for each gene. Statistical significance was determined by multiple unpaired t tests with a FDR of 1%.

### RNA isolation, RNA sequencing, and analysis:

Five mL of fresh LB medium were inoculated in triplicate with a 1:1000 dilution from overnight LB cultures of the indicated *A. baumannii* strains. Cultures were grown with shaking for 4.5 hrs when total RNA was extracted as follows. To harvest cells cultures were centrifuged and resuspended in 1 mL of TRIzol Reagent (Invitrogen). TRIzol resuspended bacteria were homogenized in a bead beater with Lysing Matrix B beads (MP Biomedical). Homogenized suspensions were collected and mixed with 200 μL of chloroform and incubated at room temperature for 2 min. Samples were then centrifuged at 4 °C for 15 min, and 650 μL of the upper aqueous phase was collected and mixed with an equal volume of 100% ethanol. RNA was then extracted using a RNeasy RNA extraction kit (Qiagen) according to manufacturer’s instructions. Purified RNA was eluted from purification columns with 53 μL nuclease free water. DNA contamination was removed using the TURBO DNase kit (Invitrogen) according to manufacturer’s instructions and RNA was stored at −80 °C. Samples were submitted to SeqCenter for rRNA depletion and sequencing. Raw paired-end reads were quality filtered using fastp (version 0.24.0) and aligned to the ATCC17978VU *A. baumannii* genome using HISAT2 (version 2.2.1) with spliced alignment disabled ^(95, 96)^. Read counts were obtained using the featureCounts function of Subread (version 2.0.8) and differential expression between experimental groups was analyzed with DESeq2 (version 1.40.2) using default parameters ^(97, 98)^. Genes were included in the analysis if at least three samples had read counts ≥10.

### qRT-PCR:

Overnight cultures of indicated *A. baumannii* strains were used to inoculate fresh LB media 1:1000 and cultured at 37 °C with shaking for 4.5 hrs when total RNA was extracted and DNase treated as above. cDNA was generated from 1 μg of extracted total RNA using the iSCRIPT cDNA synthesis kit (Bio-Rad) according to manufacturer’s instructions. cDNA template was diluted 1:50 in nuclease free water and used in qRT-PCR reactions using iQ SYBR green supermix (Bio-Rad) with the primer pairs listed in Table S2, 16S rRNA primers were used as the internal control for all qRT-PCR experiments. Indicated fold change values were calculated using ΔCT method, relative to 16S rRNA transcript abundance.

### *E. coli* 2 plasmid system:

Overnight DH5 *E. coli* cells harboring both pBAD33.1-*06475* and indicated mScarlet-I transcriptional fusion reporters were subcultured 1:50 into fresh LB medium. Subcultures were used to inoculate 100 μL of fresh LB medium 1:100 in the wells of black walled 96 well plates in triplicate with the indicated concentrations of L-arabinose to induce *06475* expression.

### Static biofilm production:

*A. baumannii* biofilm production was quantified as previously described ^(99)^. Biofilm formation was quantified in the wells of 96 well polystyrene plates. Wells were inoculated with 150 μL of a 1:20 dilution of overnight cultures of indicated *A. baumannii* strains in LB medium. Plates were grown static at 37°C for 16 hrs before cultures were removed and washed 3 times with sterile PBS, stained for 10 minutes with 150 uL of 0.1% crystal violet, and washed an addition 3 times with sterile PBS. Crystal violet stained biofilms were then dissolved in 150 μL 30% acetic acid with shaking for 10 minutes when absorbance of the resulting dissolved biofilm was measured at 570 nm. For biofilm images the above method was repeated with 1 mL of bacterial cultures in 5 mL polystyrene tubes and the resulting crystal violet biofilms were imaged upside down.

### Quantification, statistical analysis, and software:

Raw data were recorded in Microsoft Excel and imported into GraphPad Prism 10 for statistical analysis. *In vivo* images were captured and analyzed using PerkinElmer Living Image software. Figures were generated in GraphPad Prism 10 and designed in Canvas X. Data were analyzed as indicated in figure legends. Asterisks indicate the statistical significance: * p < 0.05, ** p < 0.01, *** p < 0.001, **** p < 0.0001, ns = not significant. N values, definitions of center, and dispersion and precision measurements for each experiment are reported in the figure legends.

## Supplementary Material

Supplementary Files

This is a list of supplementary files associated with this preprint. Click to download.

- AimSSupplementFinal.docx
- AimSTablesS5S11Final.xlsx

Figures S1-S6 and Tables S1-S4 and supplemental references. Excel files containing additional data too large to fit in a PDF – Table S5, untargeted metabolomic data related to Figure 3, Tables S6-S9, transposon sequencing results related to Figure 4, and tables S10 & S11, RNA sequencing results related to Figure 5.
